# Collectors on illicit collecting: Higher loyalties and other techniques of neutralization in the unlawful collecting of rare and precious orchids and antiquities

**DOI:** 10.1177/1362480615607625

**Published:** 2015-12-01

**Authors:** Simon Mackenzie, Donna Yates

**Affiliations:** University of Glasgow, UK; University of Glasgow, UK

**Keywords:** Antiquities, art crime, neutralization, orchids, regulation, trafficking, wildlife

## Abstract

Trafficking natural objects and trafficking cultural objects have been treated separately both in regulatory policy and in criminological discussion. The former is generally taken to be ‘wildlife crime’ while the latter has come to be considered under the auspices of a debate on ‘illicit art and antiquities’. In this article we study the narrative discourse of high-end collectors of orchids and antiquities. The illicit parts of these global trades are subject to this analytical divide between wildlife trafficking and art trafficking, and this has resulted in quite different regulatory structures for each of these markets. However, the trafficking routines, the types and levels of harm involved, and the supply–demand dynamics in the trafficking of orchids and antiquities are actually quite similar, and in this study we find those structural similarities reflected in substantial common ground in the way collectors talk about their role in each market. Collectors of rare and precious orchids and antiquities valorize their participation in markets that are known to be in quite considerable degree illicit, appealing to ‘higher loyalties’ such as preservation, appreciation of aesthetic beauty and cultural edification. These higher loyalties, along with other techniques of neutralization, deplete the force of law as a guide to appropriate action. We propose that the appeal to higher loyalties is difficult to categorize as a technique of neutralization in this study as it appears to be a motivational explanation for the collectors involved. The other classic techniques of neutralization are deflective, guilt and critique reducing narrative mechanisms, while higher loyalties drives illicit behaviour in collecting markets for orchids and antiquities in ways that go significantly beyond the normal definition of neutralization.

## Introduction: Morals, motives and neutralizations

Collectors lust after a contraband orchid for the same reasons connoisseurs seek a forbidden manmade masterpiece, according to prosecutor Hochman: ‘For its beauty, its rarity, its endangeredness.’

(Orrick, 1995)

Why do people buy, keep and display rare and precious ‘collectibles’? How do they talk about this social practice; and how do they explain why they do it even when it breaks the law? In this study we look for answers to these questions around legally transgressive motive, from the perspectives of collectors of two different rare and precious commodities: orchids and antiquities.

The essence of our argument is that the moral economy of collecting in these markets valorizes the practice with reference to ideals of self-realization and public service, therefore seeing both personal and social benefits where the law simply sees crime. By ‘moral economy’ (Booth, 1994; Thompson, 1971) we mean that markets are ‘grounded in a morality of fairness, justice … and shared notions of acceptable behaviour’ (Karstedt and Farrall, 2006: 1014). The personal and social processes of cultural edification within the moral economy of collecting orchids and antiquities are explained by collectors through the use of narrative tools that seem to suggest a common pool of justificatory reference. From these vocabularies of motive (Mills, 1940), operators in both collecting markets draw socially acceptable accounts to offer as explanations of why they break the law (Scott and Lyman, 1968).

Recognizing the common themes involved in this manifestation of criminal motivation is important for engaging with attempts to regulate or control the crimes of the powerful, since as we shall clearly see, some groups of people think they are above the law and not only do not feel constrained by it, but seem not to feel any guilt or shame in breaking it. This observation fits with previous studies of white-collar crime such as Benson’s investigations of ‘denying the guilty mind’ (1985; Stadler and Benson, 2012). However, while Benson sees the process as one of neutralization, we look for a slightly more fine-grained application of that term for white-collar crimes where the moral construction of the issue seems *a priori* to have removed potential feelings of guilt from the subsequent process of moral evaluation. We suggest that in these market settings an appeal to higher loyalties is a useful explanatory concept when looking for motivation, and in that respect can be seen as a deeper and more fundamental process of rationalization than the other original techniques identified by Sykes and Matza (1957), which then come to be seen as performing more ‘second-tier’ or ‘deflective’ functions in warding off critique.

The usual post-*Delinquency and Drift* interpretation of techniques of neutralization as attenuating the ‘moral bind to law’ and facilitating drift (Matza, 1964) does not seem wholly apt to help explain the illicit practices of the collectors who are the subjects of the current enquiry. They are not appropriately described as people who have a generalized commitment to conventional law-abiding norms, in respect of which their law-breaking is an occasional aberration they excuse in the moment, or situationally, but disapprove of in the abstract. Rather they are in the category of those who ‘unconditionally approve’ of the illegal act—a category that a number of commentators have differentiated from those who use techniques of neutralization, including Sykes and Matza themselves, but perhaps most explicitly Minor (1981: 300–302). For sure, collectors may value the conventional norms of legitimate society in most other parts of their lives, and they no doubt value the conventional norms of their collecting systems, in the sense of the day-to-day routines of public market and collecting/display activities. However, the key point is that this very ‘conventional’ normativity in the collecting world seems to include within it norms approving of illicit acquisition of orchids or antiquities. *In some markets, then, illegality is normatively acceptable*. If deviance from (some) legal norms is in fact conventionally normative in certain markets, our original motivational question of why people are breaking the law in these circumstances seems to merit an answer that may be framed in the language of an appeal to higher loyalties (the norms and values of the particular market), but can be done while thinking of higher loyalties primarily as a motivational driver and only secondarily, if at all, as a technique for neutralizing departure from the wider and more diffuse norm of obeying the law in all situations. Thus we come to see ‘higher loyalties’ in these markets first and foremost as motivation in a way that separates it from the other techniques of neutralization. Unlike higher loyalties, the other ‘classic’ techniques of neutralization have little explanatory power in relation to the core motivational ‘why do they do it’ question, although they do perform the useful role of helping protagonists deal with uncomfortable knowledge about the harmful effects of their actions.

The short answer to the ‘why’ question, which we will explain with supporting narrative evidence in what follows, is therefore that the collecting markets under study propagate a value system that emphasizes goals that legal regulation interferes with. Capacity to ignore or circumvent the law in such a context appears to be a feature of successful membership of these collecting groups. Put in such terms, the observations in the article sit squarely in the literature on aetiology and regulation in white-collar crime more generally (e.g. Benson and Simpson, 2009; Braithwaite and Geis, 1982; McBarnet, 2003; Pearce, 1976). The question whether that proposition is generalizable to other collecting markets in rare and precious chattels is one for further research: it seems likely.

## The study and regulation of trafficking in illicit orchids and antiquities

The illicit trafficking of cultural objects and the illicit trafficking of natural objects have become subjects of criminological focus in the usual manner of sub-disciplinary separation which divides the scholars and the scholarship of these forms of transnational crime, with effects that are in some respects unhelpful. The trafficking of cultural objects (especially antiquities) has become part of a hybrid discourse between criminology and archaeology, forming a perceived specific type of global trafficking and treated accordingly by many researchers and most international organizations: as a crime problem *sui generis* demanding regulatory responses specific to the perceived problem of ‘illicit antiquities’ (e.g. Manacorda and Chappell, 2011). Trafficking in flora and fauna, comparably, has become part of a criminological sub-discipline generally referred to as ‘wildlife trafficking’ (Wyatt, 2013), which itself is a category that is often subsumed within the ambit of ‘green criminology’ (White and Heckenberg, 2014), a much broader category focused on environmental crimes and harms but which does not include damage to cultural heritage, although some green criminologists have recognized the importance of wildlife and the environment to culture.

While the market-end dealing and collection of illicitly trafficked antiquities as a form of white collar crime has been explored by the recent work of a number of scholars (e.g. Bowman, 2008; Brodie and Bowman, 2014; Brodie et al., 2013; Chappell and Polk, 2009; Mackenzie, 2007, 2011; Mackenzie and Green, 2008; Polk, 2000) the particulars of the market-end dealing and collection of illicitly trafficked rare orchids have not been similarly assessed. We suggest that despite significant differences in how they are governed in law and researched in criminology, orchid and antiquities trafficking and collecting can be identified as having striking similarities on basic sociological levels including motivation, patterns of activity, practical ethics, geographical reach and social and cultural structures supporting economic supply and demand.

Orchidaceae is a diverse family with over 870 genera, 25,000 species and 100,000 hybrids and cultivars making it the largest flowering plant family (Swarts and Dixon, 2009). Flowers across the family are extremely varied with seemingly infinite permutations of size, shape, colour and flower clustering. Many orchids react well to hybridization, allowing for enthusiasts to create their own new orchids. Beyond some specific cases of the use of orchids in food and medicine (e.g. Subedi et al., 2013), the majority of human orchid ‘consumption’ is for enjoyment: for decoration and beauty, for an interest in botany or for collecting (Clemente Muñoz, 2009).

In orchid collecting, like antiquities collecting, rarity, uniqueness and beauty are marks of both esteem and profitability and thus demand (Clemente Muñoz, 2009; Thomas, 2006). Because of this demand, the numbers of certain types of rare or more sensitive orchids have been reduced in the wild. This has been due both to habitat loss via development and to over-collecting for the market (Swarts and Dixon, 2009). Collecting of rare wild orchids continues despite efforts to regulate the market and criminalize the activity (Flores-Palacios and Valencia-Díaz, 2007; Thomas, 2006). Many orchid species are considered threatened, endangered, extinct in the wild or completely extinct (Flores-Palacios and Valencia-Díaz, 2007; Swarts and Dixon, 2009).

The Convention on International Trade in Endangered Species of Wild Fauna and Flora (CITES) serves as the primary means to regulate the international trafficking of rare orchids. Six specific orchid species and two orchid genera are listed in Appendix I of CITES, meaning that they cannot be exported or imported for commercial purposes unless they are shown to be both artificially propagated and bear official export and import permits. This effectively bars their movement for any reason other than scientific study. The entire Orchidaceae family is included in Appendix II of CITES, meaning their movement across borders for any reason, including commercial purposes, requires official export permits. Artificially propagated orchid hybrids may qualify for a CITES exemption but even these require an exemption certificate. In addition to CITES, numerous state and local regulations bar the collection, export, import and sale of many orchid species.

Purchasing any orchids listed in Appendix I of CITES without being presented with proper paperwork attesting to the exact circumstances under which that plant or hybrid came into being is unlawful. Intentional mislabelling of non-flowering orchids and other improper export and import regulations means that even the purchase of an Appendix II orchid with proper permitting may have facilitated the smuggling of Appendix I plants.

Like orchid trafficking, antiquities trafficking is linked to destructive practices at source. Antiquities collection in its current form began in 18th- and 19th-century Europe during the time of ‘The Grand Tour’. The focus of these collectors was the glory of Egypt, Greece, Rome and West Asia; in other words the perceived progenitors of European greatness (Swenson and Mandler, 2013). Nearing the end of the 19th century, the field of archaeology split from antiquarianism and antiquities collecting to become an academic field, the focus of which was to gather information about past culture rather than to form antiquities collections (Yates, 2013). To gather this information, archaeologists require sites to be as intact as possible so that the context of the site is preserved for study. Sites ransacked for sellable goods have had their context destroyed and thus are unsuitable for archaeological study (Brodie and Renfrew, 2005; Coggins, 1970; Renfrew, 1999). Antiquities removed from their original contexts are also unsuitable for archaeological study (Brodie et al., 2000). Looted sites and looted antiquities represent an incalculable amount of lost information about the past.

Over the course of the second half of the 20th century, local and international measures were implemented in the hope of turning what continues to be a tide of looted antiquities entering the art market (O’Keefe, 1997). The basis of much of this regulation is the 1970 UNESCO Convention on the Means of Prohibiting and Preventing the Illicit Import, Export and Transfer of Ownership of Cultural Property, which implores signatories to protect sites on the ground and to effect the return of looted cultural objects (O’Keefe, 2000). The convention does not mandate specific export and import controls; rather it urges signatories to develop their own import controls, and one model for this has been exemplified in the country-to-country five year term negotiated bilateral agreements used by the USA. Beyond policy, in the early 2000s a number of high-profile cases of criminal prosecution and civil seizure have brought the destructive nature of antiquities trafficking to the attention of the public (Gerstenblith, 2007).

We can therefore contextualize the analysis that follows in this article with some base observations about these two global illicit markets, based on the brief reviews of laws and practices set out above. First, both the looting of archaeological sites and the wild collection of orchids are inherently destructive acts. Site looting causes archaeological sites to cease to exist and orchid ‘poaching’, as the practice is known, spoils fragile ecosystems. Most experts support this assertion (for antiquities see Brodie and Renfrew, 2005; Brodie et al., 2000; Chippindale and Gill, 2000; Elia, 2001; Mackenzie, 2005; Renfrew, 1999; Yates, 2014. For orchids see Clemente Muñoz, 2009; Davenport and Ndangalasi, 2003; Ferrier, 2010; Neng, 2010; Subedi et al., 2013; Swarts and Dixon, 2009). Second, the market-end demand for orchids and antiquities is what inspires a supply to be found and is the root cause of looting and poaching. This is a basic economic model that, too, is supported by available evidence and the views of experts (e.g. Gill and Chippindale, 1993; Kersel, 2008; Mackenzie, 2005; Renfrew, 1993). Third, the collectors and dealers involved are aware of the potential for illegality in the market that they engage in. This has been verified by interview research we have conducted in the antiquities trade, and the excerpted quotes of operators in the orchid trade that are reproduced in this article reveal similar patterns of knowledge.

## Accessing market narratives in collecting orchids and antiquities

Our data collection involved searching for sources in which orchid and antiquities collectors were able to express their views in their own words. Quotations from first-person interviews were collected from popular media articles that were located via targeted Google News searches for pieces related to the orchid and antiquities trades (e.g. Alberge, 2011; Bredin, 2013). In limited cases, these included statements made while in court (Pittman, 2004). A particularly interesting source of interview material for the antiquities trade was the Smithsonian Institute’s Archives of American Art, a collection of transcribed oral history interviews with key art world figures, including collectors (e.g. Fleischman, 1994; Thaw, 2007). Academic publications authored by collectors and dealers were also included (e.g. Cuno, 2014; Marks, 1998; Ortiz, 2006; White, 1998). The original context of collectors’ statements was maintained as much as possible, and all interviews used are publicly available with their sources referenced. In addition to using these public documents to gather evidence of collecting views, we have previously conducted studies that have involved interviews with collectors and dealers of antiquities, but not orchids (Mackenzie, 2005; Mackenzie and Green, 2009). These research projects included discussions of motivation and while we have not included direct quotes from the studies here, we have used the themes in these interviews and focused conversations with major collectors and dealers in the art and antiquities world to contextualize the evidence in this article when conducting our analysis.

One of the limitations of our approach to data gathering is that the statements used in this article come from a self-selecting group of orchid and antiquities collectors and dealers. They are individuals who were willing to speak to academics, the media and oral historians or were willing to pen their own articles or books. The views of less forthcoming market participants are only represented in the few cases where courtroom statements were located. Our own empirical research accessed the more hidden and private worlds of these markets to the extent such access was possible, so while the respondents in those studies are still among the most forthcoming discussants in these markets, this type of outreach provides at least some level of reassurance that we are not dealing in outlying views when we relay the public statements made by more visible collectors. Our impression is that the collectors quoted here are those who were willing to ‘raise their heads above the parapet’ and risk critical public attention, but that their perspectives are not unrepresentative of the silent majority.

## The motivation to collect

Objects came my way, and some of them unquestionably, it seems to me, because they had to do so. It is as though, imbued with the spirit of their creator, they came to me because they knew I would love them, understand them, would give them back their identity and supply them with a context in keeping with their essence, relating them to their likes.

(Ortiz, 2006: 20)

Why collect, especially when it involves choosing to participate in a criminal market? Kersel (2015) provides a review of some of the literature exploring the drive to collect antiquities. She cites Schwartz (2001: 633) in suggesting that ‘collecting has existed for as long as humans could conceptualise the idea of beauty’ and develops the analysis of the pleasure experienced through owning objects by considering it as connected to several supporting ideas. Collectors, she observes, ‘may variously view themselves as connoisseurs, heroes, public servants, saviours, tourists and harbingers of class’ (Kersel, 2015: 368). Previous studies into collecting motivations reveal the pursuit to be quite a complex field of emotional and strategic dynamics, including a means towards status legitimacy or social distinction among the nouveau riche; and ‘owner-object relations as based on domination’ (Clifford, 1985: 238; Kersel, 2015: 368), as where trophy hunting represents conquest, or looting is an act of resistance.

A large part of the idea of connoisseurship in collecting antiquities comes in the curation of a significant collection. The selective eye of the collector, combined with his or her rationale and values for choosing to create a particular type of collection, makes in effect for historical and informally scholarly statements, encapsulated and disseminated through a grouping of objects that are considered to bear a certain relation to one another. Orchid collecting is less concerned with the (re)presenting history aspect of great antiquities collections, but the emotional dynamic seems similar, experienced apparently as a sense of pleasure connected to possessing, owning, ‘saving’, enjoying and displaying rare and beautiful objects. For both types of collecting, antiquities and orchids, this pleasure can take on spiritual and other types of metaphysical dimensions, bringing to the meaning associated with collecting a sense of purpose, and responsibility to continue.

Antiquities collector Barbra Fleischman characterizes her collecting as ‘more than an intellectual pursuit’ saying that the objects have to ‘grab’ her and her husband (Muchnic, 1994). She also describes ‘the experience of a work of art’ as ‘uplifting’, which is why collecting is important to her, and that thinking about the financial value of the object is not at all the point of collecting as it ‘dilute[s] that experience’ (Fleischman, 1994).

Jean Paul Barbier-Mueller, an antiquities collector, personifies his collection by ‘engaging in a dialogue with it’ (Martin, 2013). He states that he ‘was captivated by the sensual pleasure we can experience just by gazing at an object’. Au Yon Nang Yip, an orchid collector, takes the personification a step further: ‘I treat these orchids as part of my family. They are like my wife. I live and sleep with them’ (*New Strait Times*, 1999: 19). Most collectors of antiquities and orchids report a visceral, sensual attraction to the objects.

Antiquities collector Christian Levett calls collecting ‘a compulsion to fill the gaps’ (Bredin, 2013). Compulsion is a word often used to describe both antiquities and orchid collecting. Indeed, in the 19th century the term ‘orchidelirium’ was coined to describe the compulsive behaviour of orchid collectors (Doyle, 1995). Collecting as a ‘compulsion’ set in the context of regulatory barriers to the satisfaction of that compulsion can be seen to be a fertile source of motivation for law-breaking; of the difficult pre-neutralization question of ‘will’ to offend that has been considered the weakest or at least most elusive part of Matza’s (1969) conception of ‘becoming deviant’. In a social setting characterized by this kind of strain in which the seductions of crime are unusually apparent, we can identify among the discourse of collecting what we have previously noted to be the appeal to higher loyalties. Collecting as a passion, a drive and in the conception of the famous antiquities collector George Ortiz whose quote opened this section, even as a perceived inevitability, sets up a tension between two sets of rules which compete for social adherence: on the one hand the law, and on the other the significantly socially embedded and status laden cultural goal of exotic collecting. This type of adherence to one set of (cultural) rules over another (the law) seems to be the master narrative here.

The loyalties to which high-end collectors of antiquities and orchids appeal can be appropriately summed up as a love for the things, although this raises a host of further interesting questions, including: how that type of love is manifested; how or indeed if it can be distinguished from a compulsive type of object fetishism; and where the rather less emotive financial motivation intersects with this type of desire. For example, convicted orchid smuggler Michael Kovach told the judge during his sentencing: ‘I love these plants … I did what I did without any intention of violating any laws’ (Pittman, 2004). In his case Kovach himself collected the orchid in Peru and transported it to the United States. From this love for the things comes the sense of higher loyalty, which can apparently completely erase feelings of guilt in transgressing the law. Convicted orchid smuggler George Norris also places his actions as morally separate from actual crimes: ‘[t]he hardest thing I ever did was stand there and say I was guilty to all these things. I didn’t think I was guilty of any of them’ (Grossman, 2009).

Love of orchids or ancient artefacts leads to a desire to acquire them that can sometimes manifest as a desire to ‘save them’, although there is some implicit confusion in the ‘saving’ narrative as to what the objects are being saved from. On the surface the argument is that to acquire them saves them from destruction: antiquities and rare orchids will be kept by the collectors in the best conditions to store or nurture them (Merryman, 2000). This is therefore an argument in favour of preservation. There is, however, another type of saving which is less obvious in the discourse but which is a fair interpretation of the sentiment connecting many collecting reports: the collector saves the objects from obscurity. This is therefore an argument in favour of display rather than preservation as such. Antiquities and orchids may in fact be perfectly well preserved in their original findspots around the world, in deep jungles for example, although this is not always the case given infrastructural development, environmental disaster, war and several other types of threats to their integrity. Yet even if they did not need the collector to save them from potential destruction, there is a strong sense in the collecting narrative that they deserve to be appreciated—they deserve to be loved—and in this sense the collector offers salvation.

Statements made by antiquities collector George Ortiz (2006: 25) exemplify the saving narrative: ‘[i]n these cases, horrible as the destruction is, the market is an outlet that saves some of what may be found’. Similarly, some orchid collectors cast deforestation as a generally lamentable prospect with a practical upside in being a vector for discovery. Mike Serpa, an orchid cultivator speaking on deforestation, says, ‘[y]ou find things that people weren’t able to get in to. They’re discovering [animal and plant life] in China and Vietnam that they previously thought were gone’ (Orrick, 1995).

The ‘saving’ narrative casts the actions of the market-end actor not only as positive, but heroic. As habitats and sites are destroyed, usually (at least within this narrative) by forces unrelated to the market such as war or encroaching development, collectors save so-called ‘orphaned’ antiquities and orchids: they care for them, conserve them and protect them for the good of all humanity. Laws restricting transnational movement are considered to be detrimental to the survival of orchids and antiquities; violating them is a moral prerogative. Antiquities collector George Ortiz (2006: 15) is clear about his belief in this narrative, writing ‘I believe that artefacts and art are universal heritage of mankind; that collecting is both ethical and fundamental to saving the past’ and that ‘[c]ollecting is historically responsible for saving the past’ (2006: 16). This is mirrored in the words of orchid grower Jerry Fisher: ‘[c]ollectors aren’t always the bad guys they’re made out to be. If it wasn’t for collectors, we wouldn’t know anything about all these flowers that exist’ and ‘[t]here are a lot of species in greenhouses that don’t exist in the wild any more due to habitat loss’ (Doyle, 1995).

Both antiquities and orchid collectors cite specific non-looting/poaching threats that they are saving their objects from. ‘As collectors’, says Shelby White (1998: 170) ‘we believe we are preserving and expanding knowledge of the past’, and she implies objects not smuggled out of Afghanistan in the 1960s would likely have been destroyed during conflict. She speculates that an object in her collection was possibly sold by Afghans who needed the money to fight off the Russian invasion (White, 1998: 172). James Cuno (2014), director of the J Paul Getty museum and noted spokesperson for the antiquities trade, states in respect of conflict in Syria and Iraq that ‘ISIS will destroy everything in its path’, while calling for an open-door policy for the free movement of antiquities in the market. Don Herman, then president of the American Orchid Society, describes forest fires in Brazil in 1981: ‘[w]hat they were doing was burning jungle, burning forests, and in every one of those forests they were killing all the orchids that were in there’ (Ferrell, 1995). For orchid collectors, the image of the burning rainforest generates their moral prerogative to discover, collect and propagate rare orchid species before they are gone. James Rose, an orchid dealer, conveys this sentiment: ‘If I can’t get it, I can’t grow it … and, man, in probably five more years it’ll be gone anyway’ (Ferrell, 1995). The parallel to the ‘saving’ narrative of antiquities collectors is clear.

Collectors of both commodities, while discussing their wish to save the objects, often cite some degree of public access. Through the act of saving these objects, they do a moral good by allowing them to be studied or just seen. For example White (1998: 172) casts her role as caring for unloved antiquities until they inevitably end up on public display: ‘[w]e consider ourselves preservers of the objects that we have acquired. We have, until such time as our collection will be given to a museum, the obligation to care for the objects’. Until then, ‘[a]s collectors, we take pride in knowing that the works of art in our collection are viewed by visitors to the museums to which we continually loan them’ (1998: 170). Antiquities dealer Peter Marks (1998: 120) says that seeing objects he has handled on display in a museum is ‘enormously gratifying’ and that ‘[i]t gives us a sense that we have contributed something to the visual commonweal’. Antiquities collector Christian Levett agrees: ‘To walk around the museum and see other people enjoying the pieces, surreptitiously listening to their comments, will be tremendously exciting’ (Alberge, 2011). Rose, speaking about illicit Vietnamese orchids found for sale in Japan, says the orchids ‘wouldn’t be there in North Vietnam, but we can’t go there anyway. And they are out in the trade now, so every orchid guy can have one. So actually, we’re better off, probably’ (Ferrell, 1995). In other words the orchids are better off being preserved and studied in the greenhouses of the world rather than inaccessible to all in a remote jungle.

Approval by formal academic experts is also cited under this narrative as a form of validation of collecting activities. Shelby White (1998: 170) believes that she and her husband ‘have built a collection of interest to scholars as well as the general public’. Barbara Fleischman (1994) says the same thing: ‘[a] lot of scholars come to our house from Germany, France, England, Italy—all over the world—Greece’, going on to say that she publishes her collection fully so that scholars will have something to study. Orchid collector Au Yon Nang Nip states that his orchids were open for the viewing of writers and experts, but not for his own glory. Speaking of a particular book about his orchid collection, he says ‘Not only did I not profit from the publication of the book, my name was mentioned only fleetingly’ (*New Strait Times*, 1999: 19).

Cultural edification of the public or scholars is not the only focus of this narrative. Many collectors acknowledge that they are in it for personal reasons of cultural capital (Bourdieu, 1986). Au Yon Nang Nip, talking about collecting orchids in the past, says: ‘[i]t was a status symbol then’ (*New Strait Times*, 1999: 19). Eugene Thaw (2007), speaking about the visibility of his name on antiquities that he donated to the Met Museum, says:

And if you go into the Oriental wing upstairs off the big open hall there, off the balcony, the first cases that you come to on the wall are a lot of these pieces which I gave them, and they’re listed as Thaw Collection.

Michal Kovach, an orchid collector convicted of smuggling, is quoted as having said ‘I wish I could have a plant named for me’ (Pittman, 2004). Says Bill Bergenstrom, an orchid dealer: ‘[i]f you can get a plant that nobody in the world has but you, you have done something that has put you way above everybody else’ (Ferrell, 1995).

It is interesting to consider whether in a competitive collecting marketplace, where norms of acquisition support purchases of illicit as well as licit items, and where buyers know they are breaking the law in making illicit acquisitions, the thrill of transgressing a legal norm can bring illicit collecting within the ambit of the literature on forms of ‘edgework’ (Lyng, 1990, 2005). Operating beyond the law, where the rarest and most prized items are those most likely to be illicitly obtained, does illegality heighten the buzz in the emotional dynamics of collecting in ways that are counterproductive to attempts at regulatory control? It certainly seems that the risk involved in collecting can be part of the attraction. Antiquities collector Michael Steinhard, speaking on the legal dangers of collecting, says: ‘[i]t is a little bit dangerous, but that is what makes it exciting. But life is filled with risks, isn’t it?’ (Coolidge, 2006). Carlo A Balistreieri, an attorney for the American Orchid Society agrees: ‘[t]here are people who are kind of romanced by the idea that a thing’s been smuggled in … who want to get it no matter what’ (Ferrell, 1995). That the thrill of the chase can be a significant seduction for collectors of rare and precious goods is, for example, reflected in the title of one of the several collecting books published by Thomas Hoving (1975), previously director of the Metropolitan Museum in New York: *The Chase, the Capture*.

## The moral scaffolding of appeal to higher loyalties

Orchid collector Douglas Thompson, speaking about doing business with orchid smuggler Harto Kolopaking, states ‘[i]t’s not like he is some secret mastermind. Everybody knows what he’s been doing. I have half a dozen of his plants. They were ripped off trees and arrived in boxes’ (Orrick, 1995). Although ‘everybody knows’ what is going on, collectors in these fields are certainly aware that their moral construction of the issues is at odds with the law in key respects, and that this is changing both public perception of their roles and necessitating changes in the levels of visibility with which they can perform them. Peter Marks (1998: 122) notes:

The moral world of the art dealer and the market in antiquities have recently come under attack. Previously, dealing had been generally considered, if not a noble profession, at least a glamorous line of work, even an enviable one, to those who understand the pleasure of living with works of art.

Shelby White laments: ‘[t]he unfortunate thing we’re seeing now is that other collectors are not going to show their collections because they don’t want problems. So you’re seeing a lot more secrecy than 15 years ago’ (Taylor, 2007).

If appeal to higher loyalties is the ‘master narrative’ among collectors of rare orchids and antiquities, it is supported by other subsidiary techniques of neutralization which do the job of filling out a social story that repels criticism and casts this type of collecting as a ‘good thing’. These subsidiary techniques appear to be employed in a context where participation in law-breaking is not impossible for collectors to discern and which, due to the higher loyalties involved, they do not feel merits apology. Whether therefore they are, in this context, ‘neutralizations’ is open to question: they do not seem here to ‘precede deviant behavior and make deviant behavior possible’ (Sykes and Matza, 1957: 666). The techniques outlined below look in the present study rather more like ‘justifications … commonly described as rationalisations … viewed as following deviant behavior and as protecting the individual from self-blame and the blame of others after the act’ (1957: 666).

Denial of the victim is evident where developing countries are cast by collectors as compromised in their victimization by their own failure to protect remote sites, leaving orchids and antiquities easy to steal; and this argument of a lack of adequate protection also features in arguments against repatriation of stolen artefacts (Cuno, 2014).

Due to the obscuring routines of these markets in failing to transmit clear provenance information along chains of supply, it may not be entirely clear to collectors whether in fact the goods they are buying are illicit. This injection of reasonable doubt into the process supports the denial of responsibility. Even if they are illicit, they may be the product of less egregious acts than deliberate and destructive looting. ‘While some objects appear on the market as a direct result of theft, sites are disrupted for a variety of reasons’, says antiquities collector Shelby White (1998: 171) and she goes on to suggest her antiquities may have come from dam building, agriculture and other chance finds. Christian Levitt states that ‘[a]part from the fact that a large number of items do have lengthy provenance, many non-looted objects genuinely don’t have a paper trail with them’ (O’Sullivan, 2011). In the case of antiquities this is highly unlikely and for protected orchids, this is impossible. Ray Rand, speaking to journalist Phyllis Orrick (1995) says he assumed that any orchid that made it to him was legal because it would have had to go through numerous checkpoints. Orrick notes that Rand ‘clung to’ this assumption even when he was not presented with the permits required by CITES. Rand’s denial of responsibility bears direct comparison to the purported delegation of responsibility to others which has been observed in the wider literature on white-collar crimes and was, for example, one of Jeffrey Skilling’s much remarked upon defences in the Enron inquiry and subsequent prosecutions: ‘I am not an accountant’ (Knapp, 2013: 14), in the context of suggesting that he relied on others and could not be held responsible if he had placed his trust and judgement in the hands of purported experts whom he presumed to be competent and alert. So, just as we should expect, these techniques of neutralization take on particular formulations structured by the markets in which they are used, while retaining an overall generalizable format in their more abstracted versions (Benson, 1985).

The denial of responsibility is also made easier due to the geographical, and associated moral, distance of the act of purchase from the ‘scene of the crime’ (Mackenzie, 2007). By the time the orchid or antiquity reaches the collector with an offer to purchase, the harm has already been done. Thus the causal link is denied between the market-end demand for either antiquities or orchids and the destructive forces of archaeological looting or orchid poaching. The denial of injury also features in the discourse of the two markets. The harmful effects of looting or unlawful taking of antiquities and orchids is considered over-stated. Cambodian antiquities collector Douglas Latchford claims ‘[t]he trade is not what the media has made it out to be … It is true that some of the temples there had pieces stolen, but it wasn’t on a large scale’ (Parkhouse, 2010). Mirroring this argument, John Atwood (quoted in Orrick, 1995) argues that too big a deal is made of orchid habitat loss: ‘[o]rchids are not jaguars. They produce an awful lot of seeds. They can blow three or four miles away. We think of them as rare plants, but it’s just that we can’t find them’. Orchid collector Douglas Thompson goes so far as to call CITES ‘government propaganda’ (Orrick, 1995). Shelby White promises: ‘[i]f it turns out there is something I shouldn’t have bought, I will act appropriately’ (Eakin and Kennedy, 2005) thus dismissing the idea that the looting of an artefact has caused irreparable damage, and instead asserting that any mistake she has made is minor and fixable. Orchid distributer Ray Rand indicates that wild orchid collection is not destructive: ‘[i]t’s not like you send a guy into the jungle with a sack and a machete’ (Orrick, 1995). In reality, that is exactly what you do. Orchid grower Mike Serpa calls the idea that over-collection of orchids causes extinction ‘bullshit’ and blames the loss of orchid species on development (Orrick, 1995). Collectors can therefore be straightforward and unapologetic when they consider the likely provenance of their collections. Douglas Latchford, when he was asked where an object in his collection came from, responded ‘[t]he ground’ (Parkhouse, 2010). Looted artefacts are not thought to be stolen in the ‘actual’ or ‘traditional’ sense of the word (Hawkins et al., 1995: 52; Pearlstein, 1996: 128). ‘Like many other collectors’, says Shelby White (1998: 173), ‘we make a distinction between “stolen” objects and those that are “unknown and unfound”’.

## Conclusion

Collectors of rare and precious goods have an attachment to certain loyalties perceived by them to be higher than the law. Appeal to higher loyalties is one of the more ambivalent and quixotic among the techniques of neutralization, since in bringing to the fore conflict in the individual between one set of rules (usually moral) and another (the law), it becomes difficult sometimes to say that an individual is neutralizing guilt for legal disobedience as much as they are following a passion, in our case for collecting, which in its seductions erases all traces of the legal norm as a perceived proper reference point for guiding action. As a technique of neutralization, the appeal to higher loyalties can be thought to follow the format ‘yes I know it is wrong, but…’ followed by for example ‘I was doing it for my friends’:

the delinquent does not necessarily repudiate the imperatives of the dominant normative system, despite his failure to follow them … Rather [he sees] himself as caught up in a dilemma that must be resolved, unfortunately, at the cost of violating the law.

(Sykes and Matza, 1957: 669)

As it seems to appear in our study, however, the ideal type of the appeal presently takes the form ‘it is not wrong because…’ rather than ‘yes I know it is wrong, but…’. Without apparent guilt or shame to overcome this is not a technique of neutralization, it is normative and justified illicit collecting in which the law is seen as wrong and insofar as the law may be thought to encapsulate, to borrow Sykes and Matza’s (1957: 669) formulation, ‘the imperatives of the dominant normative system’ these are indeed ‘repudiated’. Regulatory policies on trafficking have not yet reached a considered and consistent position on how to recognize and engage with the strain set up by the divergence of laws restricting the free cross-border movement of significant collectibles, and the higher loyalties of the collector to their perceived calling, whether that be ancient artefacts, orchids or perhaps something else.
